# Investigating awareness, fear and control associated with norovirus and other pathogens and pollutants using best–worst scaling

**DOI:** 10.1038/s41598-021-90704-7

**Published:** 2021-05-27

**Authors:** Kata Farkas, Emma Green, Dan Rigby, Paul Cross, Sean Tyrrel, Shelagh K. Malham, David L. Jones

**Affiliations:** 1 School of Natural Sciences, Bangor University, Deiniol Road, Bangor, Gwynedd, LL57 2UW UK; 2 School of Ocean Sciences, Bangor University, Menai Bridge, Anglesey, LL53 5AB UK; 3 Department of Economics, University of Manchester, Oxford Road, Manchester, M13 9PL UK; 4 School of Water, Energy and Environment, Cranfield University, Cranfield, MK43 0AL UK; 5 UWA Oceans Institute, The University of Western Australia, Perth, WA 6009 Australia; 6 Marine Centre Wales, Menai Bridge, Anglesey, LL59 5AB UK

**Keywords:** Policy and public health in microbiology, Virology, Environmental social sciences, Risk factors

## Abstract

Pollutants found in the water and air environment represent an ever-growing threat to human health. Contact with some air-, water- and foodborne pathogens (e.g. norovirus) results in gastrointestinal diseases and outbreaks. For future risk mitigation, we aimed to measure people’s awareness of waterborne and foodborne norovirus relative to other environment-associated pollutants (e.g. pesticides, bioaerosols, antibiotic resistant bacteria) and well-known risks (e.g. diabetes, dementia, terrorist attack). We used an online survey, which included a best–worst scaling component to elicit personal levels of control and fear prompted by norovirus relative to 15 other risks. There was a negative correlation between levels of fear vs. control for all 16 measured risks. Perceived infection control levels were higher amongst women compared to men and correlated with age and the level of qualification in both groups. Participants who had sought advice regarding the symptoms caused by norovirus appeared to have more control over the risks. Norovirus is associated with high levels of fear, however, the levels of control over it is low compared to other foodborne illnesses, e.g. *Salmonella*. Addressing this deficit in the public’s understanding of how to control exposure to the pathogen in an important health need.

## Introduction

Increased agricultural and industrial activities alongside progressive urbanization have caused the emergence and spread of a wide range of pollutants in the environment, leading to global health threats. For example, air pollutants (including bioaerosols) and pesticides are leading to an increasing number of skin and respiratory syndromes^1–4^. The overuse of antibiotics in healthcare and agriculture has led to a high prevalence of antibiotic resistant bacteria (‘superbugs’) resulting in an increasing number of untreatable bacterial infections^5^.

The number of infections and outbreaks associated with gastrointestinal symptoms are also on the rise^6–9^. Among these pathogens, norovirus (often known as ‘winter vomiting bug’) is the most common cause of non-bacterial gastroenteritis globally^10,11^. Norovirus is highly infectious, spreads rapidly and hence is responsible for outbreaks leading to the temporary closure of public places (e.g. schools, restaurants and hospitals). The symptoms, including diarrhea, vomiting, nausea, fever and abdominal pain, are usually mild and resolve in 2–5 days, however, in some cases, the infection can be life threatening^10^. The estimated annual number of cases is 685 million with approximately 200,000 deaths worldwide^10,12^ with a total of US$4.2 billion in direct health system costs and US$60.3 billion in societal costs^13^.

The main route of transmission is direct (person-to-person) contact, however, the number of cases associated with consumption of contaminated food and water is increasing^14,15^. Norovirus can be found at high concentrations in the stools of infected individuals even weeks after the symptoms are resolved^16^. As the virus is extremely resistant to traditional wastewater treatment procedures^17–19^, significant loads are discharged into the environment contaminating recreational and drinking water bodies and irrigation systems^20–22^. Furthermore, norovirus is transported in environmental waters over long distances, accumulating in sediment^23^ and taken up by filter feeder aquatic animals, such as shellfish, harvested for human consumption^24,25^. As little is known about the transport patterns and behavior of norovirus, the contamination and health risks associated with water and shellfish are hard to predict^26^.

As the virus is highly contagious and no vaccination is available, prophylactic measures are critical to the control of disease spread^27,28^. For instance, in some developed countries, information on the risks associated with norovirus and other foodborne agents are disseminated in public places (e.g. healthcare units, pharmacies, restaurant menus) and on governmental websites. Research has been done to understand the persistence and associated risks of norovirus and other pollutants in the environment^26,29–33^. Several studies have attempted to evaluate the perception of food safety and microbial contamination in general^34–37^. However, little is known about people’s awareness and perception of norovirus and its transmission^38–40^ despite the importance of such knowledge in shaping the behaviors which may reduce the spread of the disease.

This study investigated the public’s awareness and perceptions of norovirus and other environment-associated health risks. This was undertaken via analysis of data from a UK national survey. Within this survey, a choice-based approach (Best Worst Scaling; BWS)^35^ was used to determine the levels of fear and control people associated with norovirus in comparison to a set of other risks. Specifically, we investigated:

I. the level of public awareness of norovirus
II. the level of fear associated with norovirus compared to other hazards (both environment-related and more general) with established perception
III. the level of control people believed they had over norovirus compared to other hazards (both environment-related and more general) with established perception
IV. how these perceptions of control and fear varied over socio-demographic characteristics and the implications of these variations for efforts to increase awareness of norovirus and reduce the disease burden.

## Results

The survey questions and results from respondents are available at the Environmental Information Data Centre (EIDC, www.eidc.ceh.uk). https://doi.org/10.5285/0869d961-99ca-4946-9192-f35afccdda38.

### Respondents’ characteristics and experience with norovirus sources

The online survey was completed by 1006 adults between the 15th and 20th February 2018. In order to assess the awareness of people most vulnerable to water- and foodborne illnesses, individuals who had consumed bivalve shellfish and had been in contact with environmental waters within the previous year were asked to participate. After the removal of incomplete responses, those who had completed the survey so quickly as to suggest inadequate consideration of their responses (n = 194), the sample comprised 806 responses (80% of total responses). Of those 806 responders, 47% were male, 96% white, 7% were 18–24 years old, 30% were aged 24–44 years old, 38% were 45–64 years old and 25% were over 65 (Table S1). Of the respondents, 72% were parents or legal guardians with 62% having one child and 19% having two children. 47% of the children were older than 7 years (25% of the children were 7–12 and 22% were 13–18 years old).

The respondents consumed shellfish cooked in a restaurant or food outlet (89%, n = 718), at home (79%, n = 636), as ready meals (68%, n = 548) or as pickles (54%, n = 437) in the past year. Approximately one half of the sample consumed shellfish raw in restaurants (48%, n = 387) and a third consumed them raw at home (32%, n = 255). Of all respondents, 16% (n = 125) believed that they had contracted gastroenteritis (defined as “upset stomach”, see “Methods” section for details) due to shellfish consumed in a restaurant in the UK or abroad (9%, n = 70 and 5%, n = 37, respectively), takeaway in the UK or abroad (5%, n = 38 and 3%, n = 22, respectively) or as a home-cooked meal in the UK or abroad (4%, n = 34 and 0.6%, n = 5, respectively). Among the 125 respondents who believed their illness associated with consuming shellfish, most respondents started to make sure the shellfish they were eating were well cooked (75%, n = 94), told others about the experience (61%, n = 76), had a break from eating shellfish (61%, n = 76) or raw shellfish (43%, n = 54), permanently stopped eating raw shellfish of any kind (40%, n = 50) or the type that made them sick (40%, n = 50), or permanently stopped eating shellfish at home (27%, n = 34) or in a restaurant (28%, n = 35).

The majority of the respondents (87%, n = 701) had contact with UK recreational waters 4–5 times a year and 29% (n = 234) of the respondents had experienced gastroenteritis-related illness after the activity. Due to the illness, 60% (n = 140) of those people started to use hand sanitizers after recreational activity, 52% (n = 121) told others about the experience, 51% (n = 119) took a break from using recreational waters, 38% (n = 90) changed the places they visited and 24% (n = 57) changed or stopped their recreational activities. Of all 806 respondents, 22% (n = 178) had contracted gastroenteritis after nursing someone with gastroenteritis in the UK and 14% (n = 113) became ill after taking care of a sick person abroad.

### Respondents’ awareness of norovirus

Over the previous year, 40% (n = 324) of the respondents had looked for advice or guidance regarding gastroenteritis (Table 1). Of those who visited one or more of the governmental websites (National Health Services choices website, Public Health England and Wales, Health Protection Scotland, Food Standards Agency, Food Standard Scotland), 69% (n = 97) found the information useful and all would use these resources in the future. Of those who did not use governmental websites as resources, 74% (n = 136) were not aware of the resources and indicated that they would use these resources in the future. Of those respondents who did not look for advice on governmental websites (Table 1), 27% (n = 216) would use governmental websites. Of those who would not use governmental websites, 77% (n = 454) were not aware of the resources, 12% (n = 71) believed those resources would not be useful and 11% (n = 65) were not interested or would rather ask a health care professional in person.

**Table 1:** Summary on the use of information sources on gastroenteritis.

	Sought information on gastroenteritis (n = 324), n(%)	Would consider seeking information on gastroenteritis (n = 482), n(%)
Accident and Emergency Department	43 (13%)	42 (9%)
General practitioner/family doctor	121 (37%)	238 (49%)
Medical center	47 (15%)	127 (26%)
NHS 111	52 (16%)	215 (45%)
NHS Choices website	105 (32%)	188 (39%)
PHE/PHW/HPS website	45 (14%)	57 (12%)
FSA/FSS website	24 (7%)	33 (7%)
Newspapers	15 (5%)	7 (1%)
Online newspapers	22 (7%)	16 (3%)
Friends, family social media	77 (24%)	117 (24%)
Internet	112 (35%)	184 (38%)
Pharmacy	7 (2%)	43 (9%)
Other	4 (1%)	0 (0%)

NHS: National Health Service; PHE: Public Health England; PHW: Public Health Wales; HPS: Health Protection Scotland; FSA: Food Standards Agency; FSS: Food Standards Scotland.

**Figure 1:**
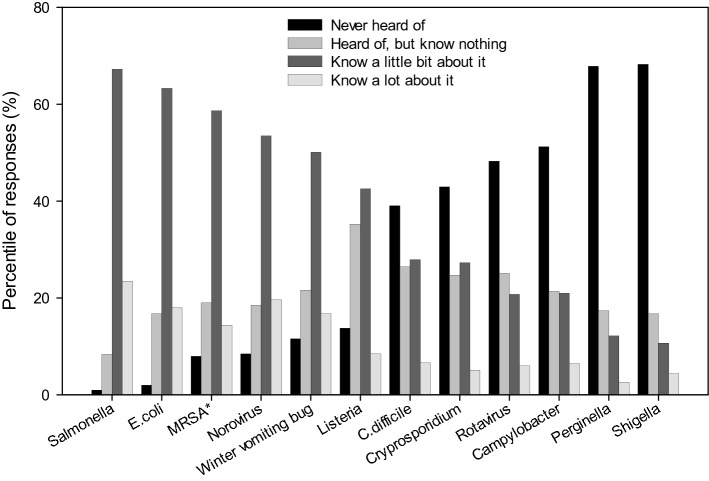
Respondents’ knowledge on pathogens causing gastroenteritis. *Methicillin-resistant *Staphylococcus aureus*.

The respondents were also asked about their knowledge of pathogens associated with gastroenteritis, including a fictive pathogen (*Perginella*) as a control. *Salmonella* was the pathogen which most people indicated they knew about followed by *E. coli* (Fig. 1)*.* Norovirus was ranked 3^rd^ (with MRSA) with 73% claiming to know a little or a lot about it. The equivalent figure for ‘winter vomiting bug’ (synonym for norovirus) was 67%. *Listeria* was ranked 6th and *Campylobacter* ranked 9th (21% said they knew a little about it, 6% claimed to know a lot) along with rotavirus, *Shigella* and the fictive pathogen, *Perginella* was reported as the least-known pathogen, with 3% of respondents claiming to know a lot about it.

### Respondents’ perceptions of fear

**Figure 2:**
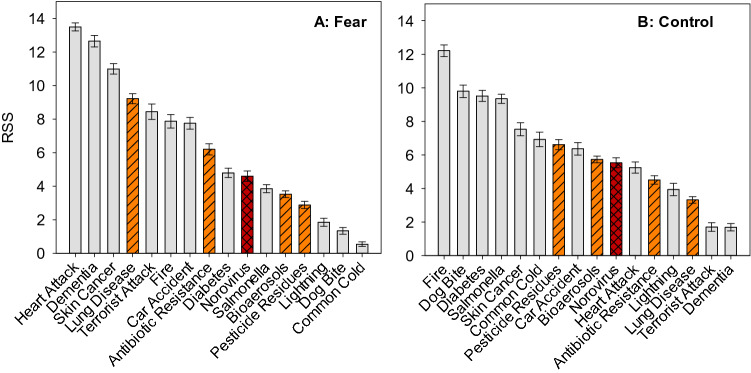
The ranking of ratio-scaled scores (RSS) relating to (**A**) fear and (**B**) control for the 16 risk items (n = 806). Error bars represent 95% confidence intervals. Red striped bar shows risk associated with norovirus and orange, crosshatched bars show other environment-associated risks.

The respondents’ ratio-scaled fear and control scores were determined based on the BWS experiment. The BWS part included 16 risks, such as norovirus, other air, food and waterborne illnesses and other hazards (Table 2). Ratio-scaled fear scores from estimation of the mixed logit model are reported in Table 3 and displayed in Fig. 2A. The most feared risk was heart attack, followed by getting dementia and becoming ill with skin cancer. The most feared environmental risk, ranked 4th, was getting lung disease from air pollution. Norovirus was ranked 10th in terms of fear, with a level of fear equal to that from diabetes. The fear associated with norovirus was 18% greater than associated with *Salmonella*, 30% greater than that from bioaerosols, and 60% greater than the fear of illness from pesticide residues. We have found no significant differences in the fear levels of people with different age, gender, education, employment status, family background, income or ethnicity.

**Table 2:** Risks used in the best worst scaling (BWS).

No	Variable name	Risk as defined in the BWS tasks
1	Terrorist attack	Being a victim of a terrorist attack
2	Dementia	Getting dementia in your lifetime
3	Car accident	Being injured in a car accident
4	Lung disease	Getting lung disease as a result of air pollution
5	Antibiotic resistance	Getting ill from bugs that are not killed by antibiotics
6	Fire	Fire at home
7	Diabetes	Getting diabetes in your lifetime
8	Heart attack	Suffering a heart attack in your lifetime
9	Pesticide residues	Becoming ill from eating substances that control pests or weeds that would remain on or in food
10	Skin cancer	Getting skin cancer in your lifetime
11	Common cold	Getting the common cold
12	Dog bite	Being bitten by a dog
13	Bioaerosols	Having breathing difficulties from inhaling spores from mold
14	*Salmonella*	Getting food poisoning from Salmonella
15	Lightning	Being struck by lightning
16	Norovirus	Catching a bug which causes nausea, vomiting, diarrhea and abdominal cramping. Sometimes called the winter vomiting bug

**Table 3:** Ratio-scaled fear and control scores (RSS, sum to 100) with 95% confidence intervals.

Risk	Fear			Control				Control (anchored)^a^			
	RSS	95% Lower	95% Upper	RSS		95% Lower	95% Upper	RSSA		95% Lower	95% Upper
Terrorist attack	8.439	7.980	8.898	1.706		1.450	1.962	− 29.595		− 32.328	− 26.863
Dementia	12.643	12.304	12.982	1.692		1.472	1.911	− 24.027		− 26.637	− 21.416
Car accident	7.750	7.398	8.103	6.367		5.997	6.737	10.793		8.297	13.289
Lung disease	9.217	8.920	9.513	3.321		3.122	3.521	− 3.831		− 6.042	− 1.621
Antibiotic resistance	6.196	5.864	6.529	4.510		4.257	4.762	− 0.063		− 2.320	2.195
Fire	7.869	7.466	8.273	12.210		11.860	12.559	37.436		34.953	39.919
Diabetes	4.794	4.520	5.067	9.519		9.192	9.846	21.964		19.409	24.519
Heart attack	13.496	13.256	13.735	5.249		4.920	5.579	6.809		4.314	9.303
Pesticide residues	2.882	2.668	3.096	6.616		6.312	6.921	5.571		3.137	8.006
Skin cancer	10.992	10.683	11.302	7.529		7.140	7.918	15.286		12.606	17.967
Common cold	0.548	0.409	0.687	6.926		6.495	7.356	10.262		7.321	13.202
Dog bite	1.341	1.142	1.539	9.794		9.424	10.163	18.412		15.827	20.998
Bioaerosols	3.527	3.329	3.725	5.736		5.534	5.938	3.129		0.911	5.347
Salmonella	3.857	3.617	4.097	9.348		9.075	9.622	20.007		17.456	22.557
Lightning	1.848	1.607	2.090	3.944		3.578	4.310	− 12.750		− 15.711	− 9.789
Norovirus	4.601	4.288	4.914	5.533		5.239	5.827	5.285		2.827	7.743

The control scores are presented as unanchored (RSS) and anchored (RSSA) scores.

^**a**^Zero = threshold for taking action to reduce the risk.

### Respondents’ perceptions of control

Respondents believed they had the greatest levels of control over fire at home, dog bites, developing diabetes and suffering food poisoning from *Salmonella* (Fig. 2B). Norovirus was ranked 10th with the perceived level of control very similar to that for bioaerosols (i.e. breathing difficulties from inhaling spores from mold). Developing lung disease from air pollution ranked 14th as the least controlled environmental risk. The level of control people felt they had over norovirus was 70% lower than the perceived level of control over *Salmonella* food poisoning and 20% lower that the level of control over becoming ill from pesticide residues. People believed they had far greater (72%) levels of control over acquiring diabetes than they did over norovirus.

**Figure 3:**
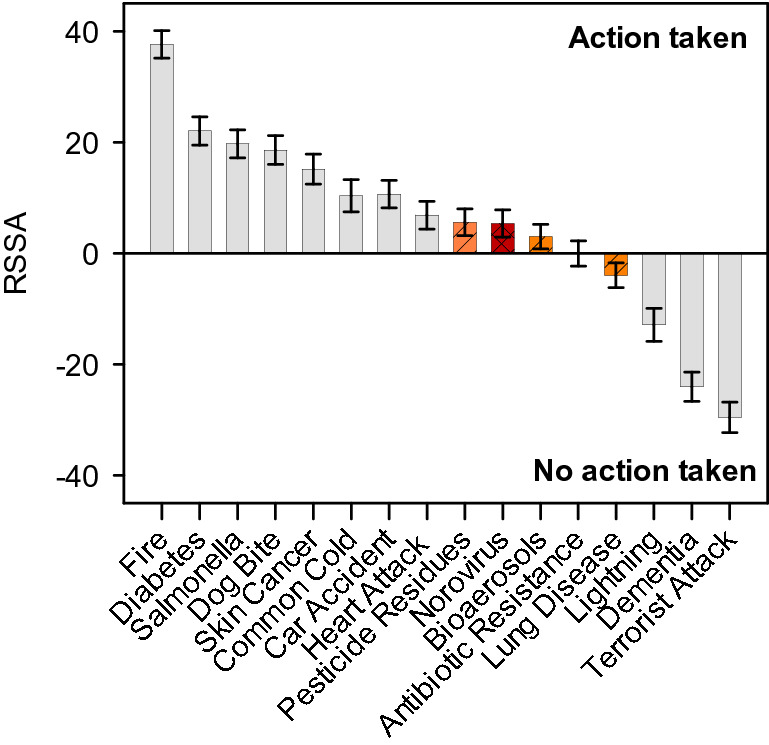
Anchored ratio-scaled control scores (RSSA). Zero = threshold for taking action to reduce the risk. The error bars represent 95% confidence intervals. Red striped bar shows risk associated with norovirus and orange, crosshatched bars show other environment-associated risks.

The consideration thus far has been relative—the degree of fear and control has been considered in relation to other risks included in the survey. The mixed logit model was re-estimated incorporating the information on which risks respondents took action to lower their risk (i.e. they exerted some control) with threshold normalized to zero (Fig. 3; Table 3). The anchored results indicate that norovirus is one of the risks people typically think they are able to control, as they do with illness from pesticide residues and bioaerosols. The marked difference in perceived control between norovirus and both diabetes and *Salmonella* is present in the anchored (RSSA) results. Getting dementia or developing lung disease are the two illness risks for which people did not take actions to lower their risk and hence scored below the zero threshold—along with ‘events’, such as lightning strike and terrorist attack.

The results reported thus far used the mean fear and control scores for each risk, generated by estimation of the mixed logit model. The model also yielded individual level estimates, conditional on their control BWS choice data and the population parameters. These estimates allowed us to consider sample level heterogeneity and systematic differences in perceptions of control, over observable characteristics. This revealed differences between age groups, genders, education levels and between those who had, and had not, sought advice on gastroenteritis (Figure S2). The analysis showed no trends or significant differences in the perception of individuals in different ethnic groups, employment status, having children or previously experiencing water- or shellfish-borne gastroenteritis.

Females perceived themselves to have higher levels of control than males for risks including norovirus, *Salmonella*, getting diabetes, illness from pesticide residues, skin cancer and illness from bioaerosols. People aged 18–24 perceived themselves to have lower control over suffering a heart attack in their lifetime than those aged 45–64.

All risks were considered more controllable among the higher educated population. The difference was significant for most risk items, except fire at home, getting the common cold, being bitten by a dog and being struck by lightning. Respondents who had sought advice on gastroenteritis believed they had more control on all the risks than those who did not. The differences were significant except for fire at home, getting diabetes, suffering a heart attack, getting skin cancer and getting breathing difficulties due to bioaerosols.

### Fear vs. control

**Figure 4:**
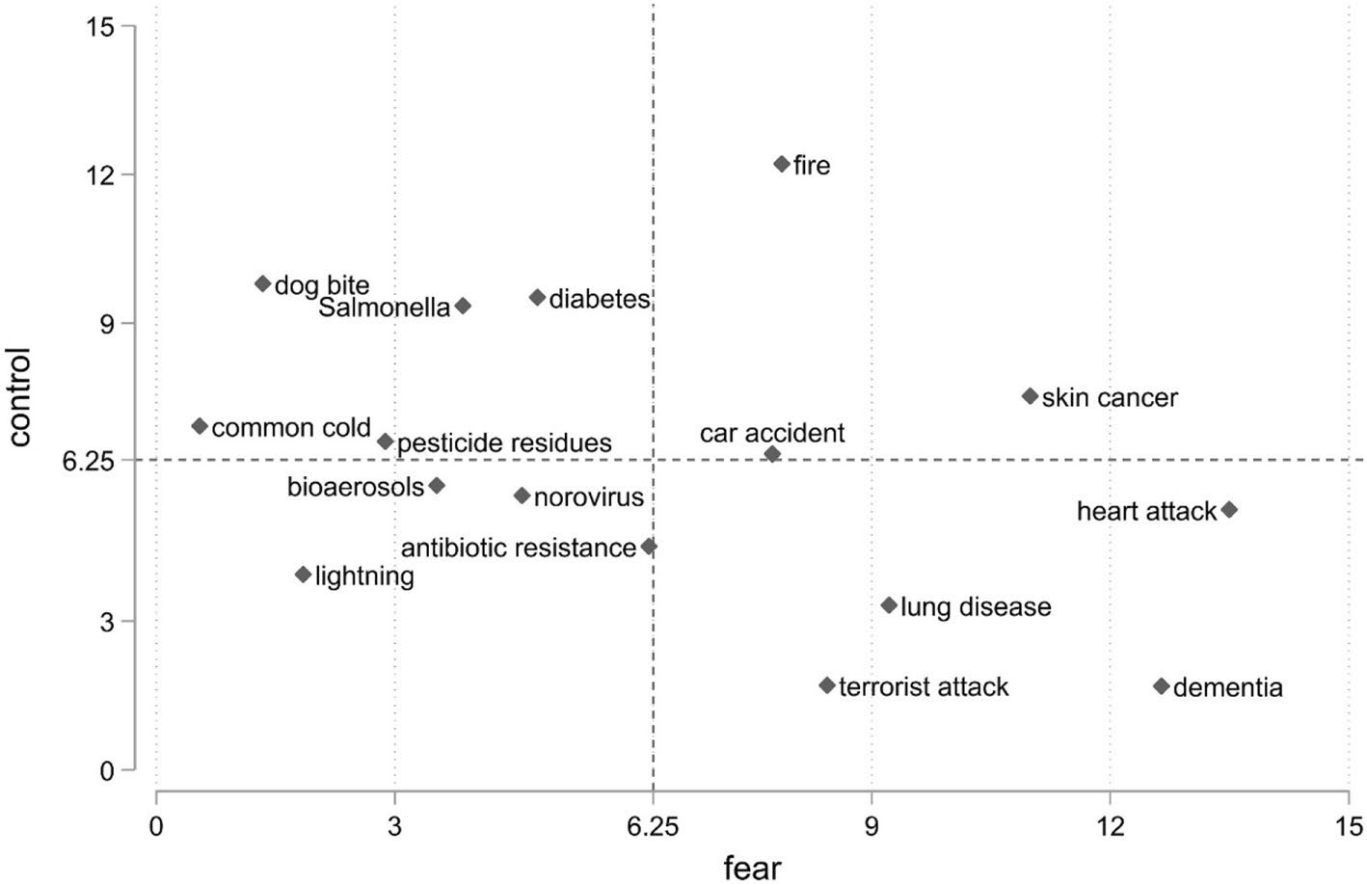
Rescaled (0–100) mean fear and control ratio-scaled scores for the 16 risk items (n = 806) in 2-dimensional space. The scaling of scores was changed to sum up to 100 with the mean of 6.25.

The results from the fear and (unanchored) control models are combined in Fig. 4 which shows the ratio-scaled fear and control scores (from Table 3). The re-scaling sums the scores to 100 and the axes represent mean levels of fear (and control) which, with 16 hazards included, is equal to score 6.25 (Fig. 4). Hence, risks ‘north’ of the x-axis indicate above average levels of control, and those ‘east’ of the y-axis indicate above average levels of fear. There was a negative correlation between the risk dimensions with higher levels of fear associated with lower levels of control. Risks, such as dementia, terrorist attack, having a heart attack or developing lung disease from air pollution, were associated with relatively high levels of fear and relatively low levels of control.

*Salmonella* was characterized by relatively low fear levels and high levels of control. Illness from bioaerosols and pesticide residues were associated with similar levels of fear as *Salmonella*, but significantly lower levels of control. Norovirus generated higher levels of fear and less control than these three hazards.

## Discussion

This study evaluated the public’s level of awareness of norovirus infection in relation to both environmental and general risks using an online survey. In order to identify the most vulnerable populations, only people regularly exposed to environmental risks (i.e. recreational water users and shellfish eaters) were included. It is estimated that 51% of the UK population consumes shellfish^41^, suggesting that the survey covered a great proportion of the population. However, there is no data available on people’s recreational activities.

Our study revealed that 28% of the respondents had gastroenteritis-related symptoms after using recreational water. Jones et al.^42^ have also found that 20% of people had diarrhea, 12% had nausea and 4% vomited up to three weeks after exposure to recreational water at Swansea, UK. In an UK-wide survey, Fleisher et al.^43^ also found that 14.9% of participants had gastroenteritis after bathing in natural waters. In both cases, the rates of illnesses were significantly higher among people exposed to water than in the control groups suggesting strong association between bathing and developing symptoms.

Our study revealed that participants had gastroenteritis after being in direct contact with a sick person (22%) and eating shellfish (16%), suggesting that these routes may also be significant routes of gastroenteritis transmission. Becoming ill from shellfish or recreational activities caused significant changes in people’s behavior; most people started to take precautions after being sick, such as increased use of hand sanitizers or no longer eating raw or lightly cooked shellfish.

The survey revealed the resources people used, or would use, to learn more about gastrointestinal diseases. We found, that although detailed information on the transmission and prevention of norovirus, and other environment-associated risks, is available on governmental websites, 74% of respondents were not aware of those resources. Understanding how and why people (fail to) become aware of the availability of such resources, could improve reduction and management of the risks. Improved understanding of those processes would be particularly valuable in relation to those groups most vulnerable to the risks.

Many respondents claimed to have good knowledge of some of the most common food- and waterborne pathogens, e.g. *Salmonella* and norovirus (Fig. 1). Their claimed levels of knowledge regarding norovirus and its synonym, ‘winter vomiting bug’, were very similar, suggesting that both terms are familiar to people. Norovirus is responsible for approx. 600,000 to one million cases each year in the UK^44^, hence the recognition of the pathogen was expected. Participants claimed to have far less knowledge on *Listeria*, a food pathogen with the most severe typical impact on health (a typical loss per case of 4.03 Quality Adjusted Life Years (QALYs) compared to 0.67 for norovirus^45^. Participants also claimed little knowledge of *Campylobacter*, even though this pathogen is the most common foodborne pathogen in the UK with over 300,000 cases and the highest annual burden on the economy (£424.4 M), followed by norovirus (£248.5 M) and *Salmonella* (£143.9 M)^45^ . Furthermore, rotavirus was not recognized by 48% of respondents. Rotavirus had been the most common cause of gastroenteritis among children under five years old until the vaccination introduced in 2013, which lowered the number of cases requiring hospitalization by 83%^46^. A similar decline in cases of *Salmonella* was observed between 2000 and 2017 due to improved legislation, food safety advice, and an industry-led flock vaccination program^47^, however, the number of lab-confirmed cases is still approx. 9000/year^48^. As all these foodborne pathogens cause mild gastroenteritis in the majority of cases, people may not be familiar with the different causative agents, and may believe their symptoms are caused by a pathogen they are familiar with (e.g. *Salmonella*, norovirus). Therefore, more information should be disseminated for people on the different kinds of pathogens. With that knowledge, people would be better able to assess their symptoms and more likely to seek medical help when necessary.

The main aim of this study was to investigate perceptions of norovirus compared to both other emerging risks associated with contaminated air and water and more established risks (e.g. heart attack, hit by lightning). The results of the BWS revealed low levels of fear and control regarding norovirus and other emerging environmental health risks compared to more general hazards. A possible reason may be that some of the risks, such as norovirus or *Salmonella* infection, mostly cause mild illness that resolve without treatment within days^10,49^.

Norovirus fear levels were equal to those from diabetes. This is remarkable given that over three million people in the UK have diabetes with this number estimated to rise to 4.6 million people by 2030^50^. Norovirus prompted significantly higher levels of fear than *Salmonella,* pesticide residues and bioaerosols. This correlates with the number of cases and economic burden of the two foodborne pathogens^45^. The levels of fear from lung disease and antibiotic resistance were higher than the level of fear prompted by norovirus. In contrast, reverse association was observed in terms of control (Fig. 2; Table 3). This suggests that along with the severity of symptoms, active management (i.e. control) of a risk also influence the overall perception of the risk. With more knowledge on the precautions one can take to avoid certain hazards, the fear prompted by the hazard may be reduced. Therefore, taking into account the high number of norovirus cases each year, the lack of concern may be due to misinformation or the lack of information on the transmittance and prevention of the disease.

The results show significant gender differences in the perceived levels of control when anchored analysis was used. In general, women believed they had more control over the risks. However, many previous studies showed that women have similar or lower levels of control than men on a wide range of risks, such as being run over/burgled, stressed environment (including technological, behavioral, and land use hazards) and earthquakes^51–54^. We found the highest differences between men and women in the control of risks associated with food preparation (getting diabetes, becoming ill due to pesticides or *Salmonella*/norovirus infection) and personal hygiene and health (developing skin cancer, common cold and antibiotic resistance pathogens). In these areas, women may believe they are more informed on the risks and take measures to reduce them by for instance thoroughly washing vegetables, adequately cooking meat and cleaning and washing hands regularly^55,56^.

Our results also showed that the level of risk perception in terms of control increases with age and educational qualifications. These findings suggest that people are more aware of the risks with more experience and knowledge. We found no significant differences in the control levels of people with different employment status, family background, income or ethnicity. Many studies have explored the effect of socio-demographic factors on behavior, e.g. disaster preparedness or consumer behavior^57–59^, however, little information is available on the effect of these factors on risk perception. More targeted analysis is therefore necessary to explore the effect of socio-demographic factors on risk perception.

The BWS results suggest that those who sought advice on gastroenteritis perceived themselves to have higher levels of control over risks (related and unrelated) to the illness. That also indicates that people who sought advice are generally more aware and cautious of risks, and hence take measures to better understand and control them. As frequent exposure to messages on health and well-being have been shown to positively influence health-related behavior^60^, the targeted dissemination of information on environment-associated health risks may increase control over risk and hence reduce the number of illnesses. The information to be disseminated may include detailed information on the transmission of norovirus and options to mitigate risk of infection, e.g. hand washing, cooking shellfish and washing vegetables before consumption, good kitchen hygiene^61^. According to our analysis, men and people with low education levels believe they have the least control on environmental risks (Figure S2), hence distribution of information targeting these socio-demographic groups may be required. Our survey revealed that the information available on public health and food safety-related websites was regarded as adequate, however, many participants were not aware of these resources. Therefore, more information (including QR code and other links to official web resources) should be circulated at places where people would look for such advice, e.g. doctors’ offices, hospitals, medical centers and the health services’ websites. Albeit controversial, more information on food safety could also be placed on shellfish food packaging.

## Conclusions

In this study, we assessed the public’s perception of norovirus infection in relation to other environment-associated and general health risks. Our results suggest that the level of norovirus awareness is relatively high compared to other foodborne pathogens (e.g. *Listeria*, *Cryptosporidium*, rotavirus or *Campylobacter*) and slightly lower than the claimed knowledge on *Salmonella*, *E. coli* and MRSA. Furthermore, our BWS results suggest that the levels of fear associated with norovirus are comparable to those from diabetes. Despite the considerable levels of awareness and fear, the level of control people felt they had over norovirus was low, significantly below that for *Salmonella* or diabetes.

Given the scale of the public health burden associated with norovirus, addressing this deficit in the public’s understanding of how to control exposure to the pathogen is an important public health need. However, our study revealed some association between perceived control on norovirus and age, gender and education. Further research is therefore needed to understand the effect of sociodemographic factors and behavior on the perception of norovirus and other food- and waterborne illnesses. Our current findings suggest that different approaches are needed to disseminate information regarding transmission of the virus to increase awareness and reduce risk of infection.

## Methods

### Survey design

The online survey we designed comprised of two sections. The first section elicited demographic information (age, gender, income, qualification, ethnicity etc.). The respondents were also asked about behaviors relevant to exposure to norovirus, their knowledge of the pathogen and their use of information sources related to it. Specifically:

- Type and frequency of shellfish consumption.
- How often, and where, they come into contact with recreational waters (including rivers, lakes and seawater in the UK).
- Whether they had had contact with anyone with gastroenteritis or food/shellfish-related gastroenteritis over the past year and if so, where the shellfish was obtained.
- Whether they had sought information about gastroenteritis over the past year and if so, whether they found the information useful.
- The reason for not seeking information and where they would consider looking.
- How much they had heard about certain enteric pathogens (*Salmonella*, methicillin-resistant *Staphylococcus aureus*—MRSA, *Cryptosporidium*, norovirus, *Escherichia coli, Clostridium difficile*, *Listeria*, winter vomiting bug, *Shigella*, *Campylobacter*, rotavirus) and a fictive one (*Perginella*), which acted as a control. We used the terms ‘norovirus’ and its synonym, ‘winter vomiting bug’, separately to assess which term is better known.

The survey referred to gastroenteritis as “upset stomach” defined as onset of stomach pains that can strike quickly with force and make a person feel very sick, which typically resolves within 2–3 days with symptoms of nausea, vomiting, diarrhea and abdominal cramping. The full list of questions is detailed in the Supplementary Material.

The second part of the survey comprised the Best Worst Scaling (BWS) exercise. BWS is a choice-based survey method designed to elicit the relative importance of multiple items enabling accurate ranking. Rather than asking respondents to rank large numbers of items, they are asked to choose the ‘most’ and ‘least’ from repeated subsets of items^35^. In our study, the items were the risks considered in the study (see Table 1) and respondents were asked to choose those they feared least and most and those they had most and least control over (Figure S1). The task was repeated with varying combinations of four items.

The BWS experiment contained 16 risks, including norovirus, other air, food and waterborne illnesses and other hazards (Table 1). Some of the risks were well-established in the risk perception literature (e.g. heart attack, car accident), whereas others were more novel (e.g. antibiotic resistance, bioaerosols). The allocation of risks into sets was determined by an experimental design which varied the combinations of risk presented, the frequency with which they occur and co-occur and their position in the sets (top, middle, bottom). Respondents completed 12 tasks using each criteria (fear, control) with the risks randomly allocated.

The approach employed here resembles that of Erdem and Rigby^53^, who elicited perceptions of a set of risks in terms of fear and control—as these had been identified in the psychometric risk perception literature as dimensions on which risks could be meaningfully located. We augmented their approach by the introduction of an absolute threshold into the analysis of control levels. The BWS method, as typically employed, is a relative assessment of the items under consideration. In this case, as well as eliciting the relative degree of fear that the hazards induced, and the degree of control people perceived they had over them, we also asked, for each risk, whether the respondent ever amended their behavior to reduce the risk. We then incorporated that threshold information into the analysis of control. Further details on survey and experiment design and model specification can be found in the Supplementary Material.

### Data collection and analysis

The survey was distributed by an online marketing company (Research Now, UK). Only adults (over 18 years old) who had consumed bivalve shellfish and had been in contact with environmental waters within the previous year were included in the study. The survey was piloted on the 13th February 2018 with 100 participants. When the required number of answers was reached, the survey was paused, and survey responses were checked to ensure that sensible answers were received. Based on the responses, the skip logic was working, people saw the right responses based on their answers and the ratio of replies were what we predicted and people who did not meet the criteria were disqualified. The survey was then administered between the 15th and 20th February 2018 with 1006 participants. After the removal of incomplete responses, those who had completed the survey so quickly as to suggest inadequate consideration of their responses (n = 194), the sample comprised 806 responses.

The BWS data were analyzed via estimation of random utility models^62^ and mixed logit models^63,64^, as detailed in the Supplementary Material. Best–worst raw scores were calculated for each risk and then transformed into standardized ratio-scaled (0–100) scores (RSS) and anchored ratio-scaled scores (RSSA) incorporating threshold data using Lighthouse Studio^65^.

### Ethical statement

The ethical approval for this study was obtained from the Ethics Committee of Bangor University. The online survey was handled by Research Now, UK. All procedures performed in studies involving human participants were in accordance with the 1964 Helsinki declaration and its later amendments. This manuscript does not contain any individual person’s data in any form. The introductory text of the survey informed the participants that the survey was part of a scientific project with its aims and methods explained, and that participation was optional. Informed consent for participation was obtained by Research now, UK.

## Supplementary Information

Supplementary Information.
