# Supplementary material for: Investigating awareness, fear and control associated with norovirus and other pathogens and pollutants using best–worst scaling
Source: Sci Rep. 2021 May 27;11:11194. doi: 10.1038/s41598-021-90704-7 (PMC8160009; doi:10.1038/s41598-021-90704-7)
Supplement: Supplementary file 1 — Supplementary Information. [file 41598_2021_90704_MOESM1_ESM.docx]

Supplemental Material

Investigating awareness, fear and control associated with norovirus and other pathogens and pollutants using best-worst scaling

Kata Farkas^1,2§*^, Emma Green^1§^, Dan Rigby^3^, Paul Cross^1^, Sean Tyrrel^4^, Shelagh K Malham^2^, David L. Jones^1,5^

^1^ School of Natural Sciences, Bangor University, Deiniol Road, Bangor, Gwynedd, LL57 2UW, UK

^2^ School of Ocean Sciences, Bangor University, Menai Bridge, Anglesey, LL53 5AB, UK

^3^ Department of Economics, University of Manchester, Oxford Road, Manchester, M13 9PL, UK

^4^ School of Water, Energy and Environment, Cranfield University, MK43 0AL, UK

^5^ UWA School of Agriculture and Environment, The University of Western Australia, Perth, WA 6009, Australia

^§^ Both authors contributed equally to this work.

* Corresponding author: Kata Farkas

email: [fkata211@gmail.com](mailto:fkata211@gmail.com)

Marine Centre Wales, Menai Bridge, Anglesey, UK, LL59 5AB

Phone: +44(0)1248 382615

**List of survey questions**

Q1. What is your self-identified gender?

Q2. Please tell us your age.

Q3. What is the chief income earner’s occupation in the household?

Q4. Where do you live?

Q5. Do you eat filter feeding shellfish? By filter-feeding shellfish we mean mussels, oysters, cockles, clams and scallops.

Q6. In the last year, how often have you eaten filter-feeding shellfish in a restaurant or at home?

Q8. How often do you think that you have had contact with UK recreational waters in the last year??

Q9. Do you think you have had an upset stomach as a result of eating food (food poisoning) over the last 12 months?

Q10. Do you think that your upset stomach was caused by eating filter-feeding shellfish?

Q11. Where do you suspect that you ate the filter feeding shellfish that caused your upset stomach?

Q12. Did the fact that you became had an upset stomach after eating filter feeding shellfish lead to a change in your behaviour?

Q13. Have you had an upset stomach which made you feel very sick and lasted for 2-3 days after using UK recreational waters?

Q14. Have you had an upset stomach which made you feel very sick and lasted for 2-3 days after using recreational waters whilst abroad?

Q15. You said that you’ve had an upset stomach after having contact with recreational waters. Did this lead to a change in your behaviour?

Q16. Have you nursed someone who had an upset stomach and then became ill yourself in the UK?

Q17. Have you nursed someone who had an upset stomach and then became ill yourself whilst

Abroad?

Q18. In the last year, have you ever looked for advice or guidance about an upset stomach?

Q19. Where have you ever looked for advice or guidance about an upset stomach in the last year?

Q20. Would you consider looking at these online tools provided by the UK Government in the future?

Q21. Do you feel UK government agencies, such as the Food Standards Agency/ Food Standards Scotland, provide enough information about reasons for and prevention tips to avoid an upset stomach?

Q22. We would like to you to tell us how much you have heard about bugs that can cause upset stomachs.

Q23. To which of the following ethnic groups do you regard yourself as belonging to?

Q24. What is your highest qualification level?

Q25. How many people live in your household?

Q26. Are you a parent or legal guardian?

Q27. How many children live with you?

Q28. Finally, which of these bands does your household, pre-tax income fall into?

More details on the survey and the best-worst scaling can be found at the Environmental Information Data Centre (EIDC, [www.eidc.ceh.uk](http://www.eidc.ceh.uk)). Doi: [10.5285/0869d961-99ca-4946-9192-f35afccdda38](https://doi.org/10.5285/0869d961-99ca-4946-9192-f35afccdda38).

**Best-Worst Scaling Experimental Design and Data Analysis**

The elicitation of the perceived levels of fear and control using BWS requires an experimental design which specifies the repeated subsets of the full set of items from which respondents make their choices of ‘most’ and ‘least’. The experimental design is contingent on the specification of (i) the number of items which comprise each set, (ii) the number of sets each respondent completes and (iii) the number of versions (or blocks) of the design. Decisions regarding (i) and (ii) involve a trade-off between statistical efficiency (increasing with items per task and tasks per person) and cognitive efficiency / fatigue (eventually decreasing with items per task and tasks per person). Including more than five items per set has been found to generate little information due to fatigue or cognitive load^1^. If the researcher intends to derive individual-level scores (based on estimation of the mixed logit model) it is recommended that each item is seen at least three times by each respondent^1^. Hence, we generated an experimental design in which the 16 items were combined in 12 sets of four items. The design comprised 300 versions to which respondents were randomly assigned, to achieve balance in the co-occurrence of items and their positional frequencies (top, middle, bottom) within the sets.

The BWS data were analyzed via estimation of random utility models^2^. Underlying this approach is the conceptualization that there exists a scale of fear (and also control) on which hazards can be located by respondents. We refer to φ*_A_* as the position of hazard *A* on that scale. Respondent *n*’s unobserved fear score for hazard *A* is given by

$P_{nA}={}_{A}+\varepsilon_{nA}$ (1)

where $\varepsilon_{nA}$ is an error term, the inclusion of which creates a probabilistic rather than a deterministic choice model. The probability of person *n* choosing any pair of best-worst choices, for example hazards *A* and *D*, is given by the probability that (*P_nA_ - P_nD_*) exceeds all other *K*(*K*-1) differences within the BWS set where *K* was the number of items per set (four in this study).

The model is statistically implementable via the assumption that the error term, $\varepsilon_{nA}$, has an extreme value type I (Gumbel) distribution. This means that the probability that risks *A* and *D* were chosen as most and least feared, is given by the conditional logit formulation:

$\frac{{exp(}^{{}_{A}-_{D}})}{\sum_{b=1}^{K} \sum_{w=1}^{K} {exp}^{{}_{b}-_{w}}-K}$ (2)

Maximum likelihood estimation of (2) generates estimates of the φ fear (control) scores which maximized the likelihood of the observed pattern of Best Worst choices observed. The approach is relative, that is, the estimated fear and control scores are relative to each other on an arbitrary scale, with one hazard’s score normalized at zero for identification purposes. When an absolute threshold is incorporated in the analysis (‘anchored’ BWS) it is normalized to have a value of zero with the risks estimated relative to it (being ‘above’ or ‘below’ the threshold).

The conditional logit model in (2) does not, as specified, allow for heterogeneity amongst the sample. To accommodate such heterogeneity, we estimated mixed logit models ^3,4^ in which the fear (control) scores are assumed to be drawn from a distribution the mean and standard deviation of which are estimated. It models respondents’ control (fear) scores as a function of a higher-level model which is pooled across the sample and a lower, individual-level, model. In the higher-level model respondents’ control (fear) scores are drawn from a multivariate normal distribution, defined by mean α and covariance matrix *D*. Individual level score estimates are derived using a weighted combination of information from both the higher and lower level models. Given the non-classical means of estimation, there is no formal convergence. Instead, the estimation process is run for 8000 iterations those values are discarded (the ‘burn-in’) beyond which the values of the next 6000 iterations are retained. The estimates of the mean scores (α) are derived from the retained 8000 draws after the burn-in.

An attraction of this model is that individual-level score estimates can be retrieved. More formally, person *n*’s fear score for hazard *A* (${}_{nA}$) is drawn from a distribution with mean (${}_{A}^{*}$) and standard deviation $\sigma_{A}$. Person *n*’s fear score deviates from the sample mean (${}_{A}^{*}$) via a disturbance term, ∇ where ∇~N(0,1):

${}_{nA}={}_{A}^{*}+{\sigma_{A}}_{\mathrm{nA}}$ (3)

The estimated means of the coefficients are transformed into ratio scaled scores (RSS). The ratio scaled property allows the relative size of the perceptions of fear or control to be derived. The transformation requires that the β_i_ coefficients be re-scaled to have a mean of zero:

${RSS}_{A}=\frac{\exp{}_{A}}{(exp {}_{A}+(\lambda-1)}$ (5)

where λ was the number of items comprising each set (λ=4). These importance scores have the desirable property of being ratio-scaled, allowing a more intuitive comparison of levels of control.

The resulting RSS values convey the size of differences in perceptions of fear and control, with a hazard with an RSS value of *2x* being perceived as twice as fearful (or controlled) as a hazard with a score of  *x*.

Introducing covariates (such as gender) into the estimation deviates from the assumption of respondent preferences being drawn from a single normal distribution with mean vector α and covariance matrix *D*, with those preferences now related to covariates through a multivariate regression model.

The differences in the control scores displayed in Supplementary Figure 2 are based on individual level estimates retained post-estimation of a series of mixed logit models in which the indicated respondent characteristics (gender, age,…) have been included in the upper-level model of the hierarchical model estimated.

**References:**

1. Sawtooth. Sawtooth Software. (2020).

2. McFadden, D. Conditional Logit Analysis of Qualitative Choice Behavior. in *Frontiers In Econometrics* (ed. Zarembka, P.) 105–142 (Academic Press, 1973).

3. Train, K. *Discrete choice methods with simulation*. (Cambridge University Press, 2003).

4. McFadden, D. & Train, K. Mixed MNL models of discrete response. *J. Appl. Econom.* **15**, 447–470 (2000).

**Supplementary Table 1.** Characteristics of survey respondents.

| Classification variables | Modalities | n (%) |
| --- | --- | --- |
| Gender | Male | 381 (47.3%) |
|  | Female | 424 (52.7%) |
| Age | 18-24 | 53 (6.6%) |
|  | 25-44 | 240 (29.8%) |
|  | 45-64 | 308 (38.2%) |
|  | 65-over | 208 (25.8%) |
| Ethnicity | White (White British/Irish/Other) | 777 (96.4%) |
|  | Other | 29 (3.6%) |
| Qualification | Secondary | 249 (30.9%) |
|  | College | 271 (33.6%) |
|  | University | 286 (35.5%) |
| People in household | 1 | 104 (12.9%) |
|  | 2 | 324 (40.2%) |
|  | 3 | 150 (18.6%) |
|  | 4 | 170 (21.1%) |
|  | 5< | 58 (7.2%) |
| Parent / legal guardian | Yes | 578 (71.7%) |
|  | No | 228 (28.3%) |
| Employment status | Semi or unskilled manual worker | 113 (14.0%) |
|  | Skilled manual worker | 198 (24.6%) |
|  | Junior managerial | 175 (21.7%) |
|  | Intermediate managerial | 164 (20.3%) |
|  | Higher managerial | 71 (8.8%) |
|  | Student | 6 (0.7%) |
|  | Casual worker | 5 (0.6%) |
|  | Retired | 10 (1.2%) |
|  | Housewife/homemaker | 34 (4.2%) |
|  | Unemployed | 24 (3.0%) |
|  | Carer | 6 (0.7%) |
| Household income | Up to £6,499 | 22 (2.7%) |
|  | £6,500-£11,499 | 51 (6.3%) |
|  | £11,500-£17,499 | 62 (7.7%) |
|  | £17,500-£24,999 | 110 (13.6%) |
|  | £25,000-£34,999 | 163 (20.2%) |
|  | £35,000-£44,999 | 144 (14.1%) |
|  | £45,000-£54,999 | 81 (10.0%) |
|  | £55,000-£74,999 | 60 (7.4%) |
|  | £75,000-£99,999 | 47 (5.8%) |
|  | £100,000-£124,999 | 10 (1.2%) |
|  | £125,000-£149,999 | 10 (1.2%) |
|  | £150,000-£199,999 | 9 (1.1%) |
|  | More than £200,000 | 4 (0.5%) |


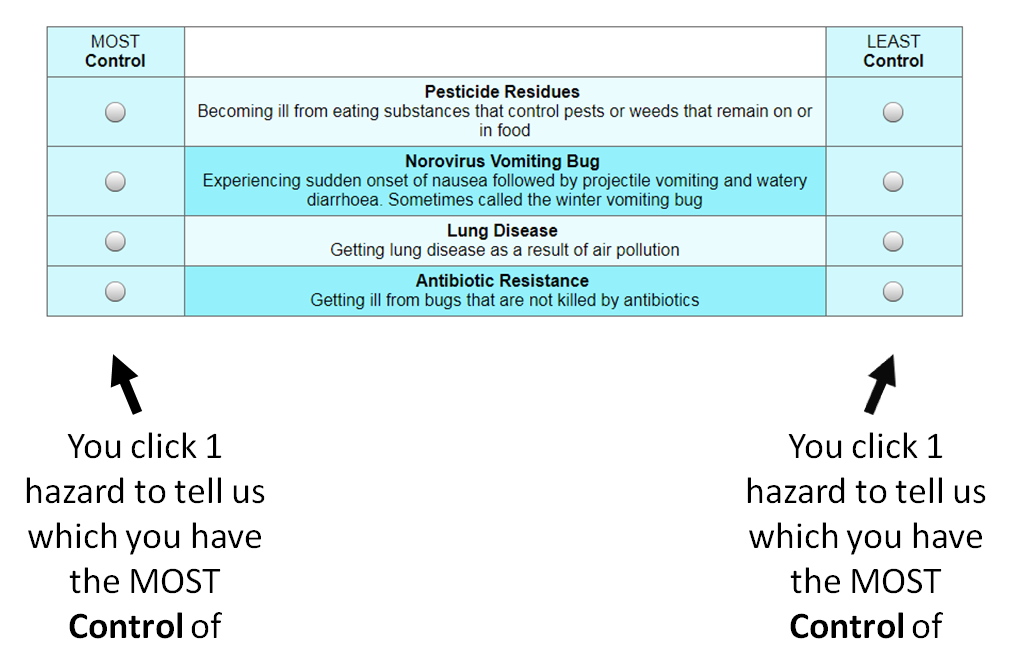


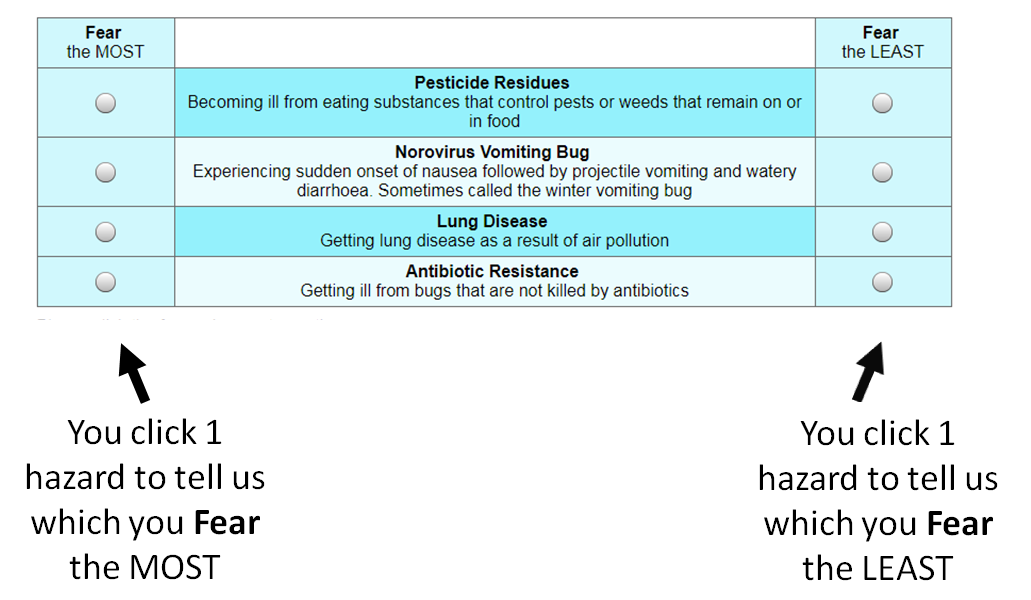


**Supplementary Figure 1.** Examples of the Best Worst Scaling (BWS) questions.


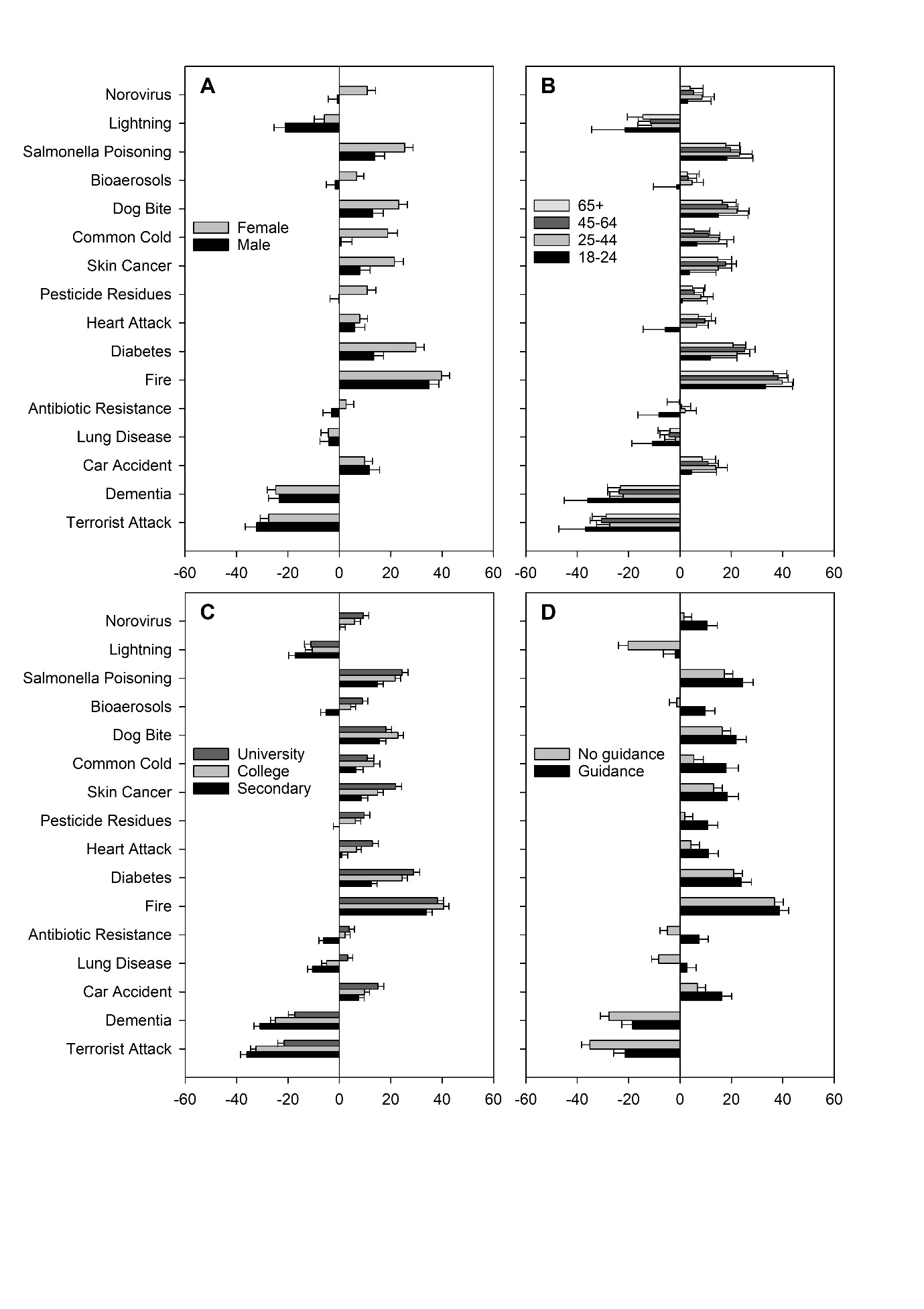


**Supplementary Figure 2.** Mean anchored control scores among A) gender, B) age groups, C) education level and D) people who sought guidance regarding gastroenteritis. The error bars represent 95% confidence intervals.
